# Cutaneous Ulcers as Initial Presentation of Localized Granulomatosis with Polyangiitis: A Case Report and Review of the Literature

**DOI:** 10.1155/2015/517025

**Published:** 2015-11-17

**Authors:** Noreen Nasir, Syed Ahsan Ali, Hafiz Mohammed Mehmood Riaz

**Affiliations:** ^1^Section of Internal Medicine, Department of Medicine, The Aga Khan University Hospital, P.O. Box 3500, Stadium Road, Karachi 74800, Pakistan; ^2^Division of Rheumatology, Section of Internal Medicine, Department of Medicine, The Aga Khan University Hospital, P.O. Box 3500, Stadium Road, Karachi 74800, Pakistan

## Abstract

*Background*. Granulomatosis with polyangiitis (GPA) is an ANCA associated small vessel vasculitis characterized by necrotizing granulomatous inflammation involving the upper and the lower respiratory tract and the kidneys. The disease has a broad clinical spectrum that ranges from limited/localized involvement of a single organ system to a generalized systemic vasculitis that affects several organs with evidence of end organ damage. Atypical forms of the disease have been recognized with or without respiratory tract involvement with a long protracted course before manifesting as generalized disease.* Case Presentation*. We describe a 57-year-old woman who presented with recurrent fever and cutaneous ulcers on her legs who was diagnosed to have granulomatosis with polyangiitis (GPA) after an extensive evaluation which excluded infectious, other vasculitides, connective tissue disease and malignant etiologies. * Conclusion*. In the absence of typical manifestations, granulomatosis with polyangiitis (GPA) is indeed a diagnostic challenge to the physician. Atypical manifestations like unexplained recurrent fever and cutaneous ulcers nevertheless call for keeping a low threshold for the diagnosis of GPA as the disease can initially present in localized form before heralding into a generalized disease.

## 1. Introduction

Granulomatosis with polyangiitis (GPA) is a small vessel vasculitis with an array of systemic manifestations. The hallmark of this disease is granulomatous inflammation involving the upper and lower respiratory tract with or without the involvement of kidneys [1]. However, the disease can involve virtually any organ in the body as generalized disease or as a limited/localized disease. In the localized disease, the inflammation is confined to eyes, ears, nose, and lungs but other organs may get involved including heart, skin, gastrointestinal tract, and the peripheral nervous system. Cutaneous vasculitis secondary to GPA can present as papules, nodules, palpable purpura, ulcers resembling pyoderma gangrenosum, or necrotizing lesions leading to gangrene [2]. There is no single lesion specifically associated with the disease. Cutaneous lesions are found in 50% of patients [3] but may be the presenting symptoms in up to 10% of cases. It is therefore of utmost importance to consider GPA in the differential diagnosis of small vessel cutaneous vasculitis so that the disease can be identified early in the clinical course and treated appropriately to prevent morbidity associated with complications arising from generalized disease [4]. Systemic GPA can cause life threatening diffuse alveolar hemorrhages as a result of necrotizing alveolar capillaritis and kidney failure necessitating hemodialysis due to rapidly progressive glomerulonephritis [5].

Granulomatosis with polyangiitis has a strong association with antineutrophil cytoplasmic antibodies (ANCAs) which characteristically have a cytoplasmic labeling pattern (C-ANCA) in immunofluorescence assays directed against proteinase-3 and myeloperoxidase [6]. These are IgG antibodies directed against primary granules of the neutrophils and the lysosomes of monocytes [7]. ANCA can be seen in 80–95% of the patients with generalized disease but in limited presentations ANCA is detectable in only 60%–80% of the cases and remains negative in 20%. Hence it is imperative to obtain tissue biopsy for histopathological evaluation as a more definitive means to diagnose this disease [6].

## 2. Case Presentation

**Figure 1:**
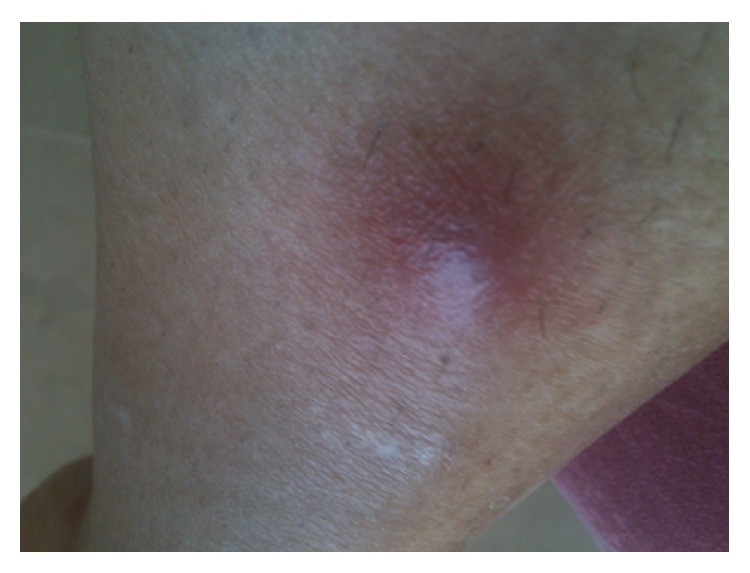
Initial lesion: erythematous nodule on right shin.

A 57-year-old woman presented to the rheumatology clinic with recurrent fever and painful ulcers on her legs since two months. Eight months prior to current presentation, she developed vertigo and decreased hearing in her left ear which she felt was “full of air” all the time. She was found to have sensorineural deafness in her left ear. Two weeks later, she developed fever with chills. A chest radiograph revealed infiltrates in her right lung and an elevated ESR. She was treated with oral antibiotics which relieved her symptoms. Then she developed gingival hypertrophy and a CT scan of the paranasal sinuses revealed subcutaneous soft tissue thickening of the right buccal area suggestive of an inflammatory process. She was treated with ibuprofen and felt better. Her past medical history was notable for hypothyroidism for 20 years and she as well was maintained on 50 mcg of thyroxin daily. She had undergone hysterectomy for uterine fibroids 15 years ago. She reported no recent travel history. Of note, she revealed contact with sick animals. She nursed her paralyzed pet dog and also had cats. She was treated with antibiotics yet continued to spike fever and came to our center for comprehensive evaluation. On examination, she appeared weak and ill. The temperature was 37.5°C, the blood pressure 139/69 mm Hg, the pulse 78 beats per minute, and the respiratory rate 16 breaths per minute, with 99% oxygen saturation at room air. Erythematous tender nodules were noticed on bilateral lower extremities (Figure 1).

**Figure 2:**
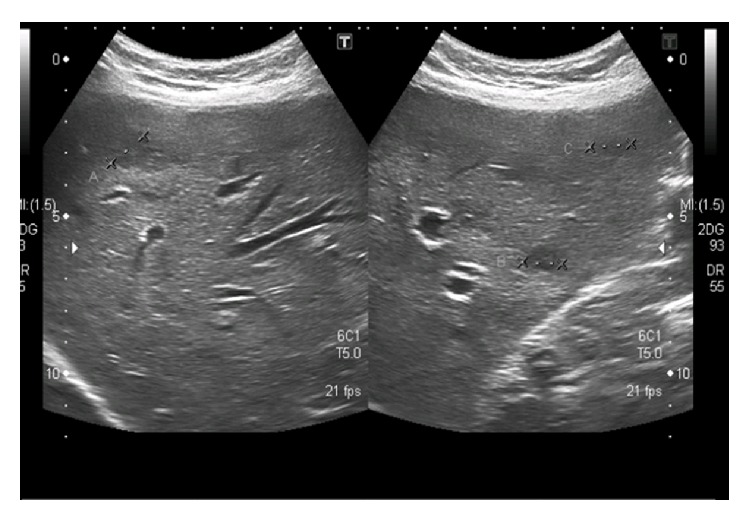
Ultrasound upper abdomen. Multiple hypoechoic tiny lesions on both lobes, few of them cystic in appearance.

**Figure 3:**
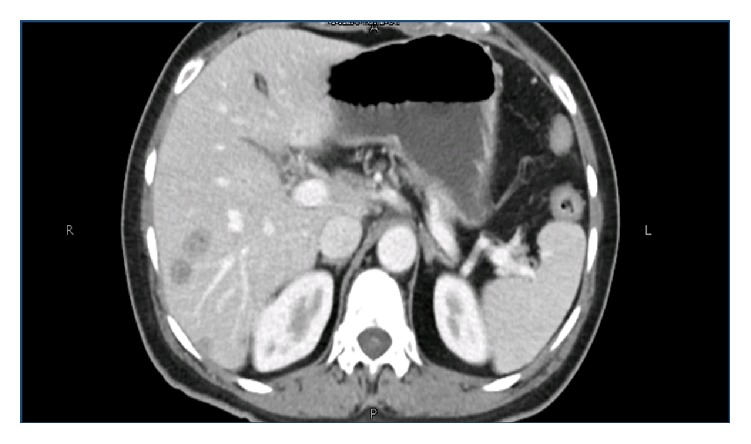
CT abdomen with contrast: multiple hypoattenuating lesions in the liver.

The remainder of the examination was unremarkable. Her complete blood count revealed normocytic, normochromic anemia with normal white cell and platelet count. Her biochemistry showed normal kidney and liver function. No active sediment was present on urinalysis. Her three sets of blood cultures and one urine culture reported no growth. The test for latent tuberculosis by interferon gamma release assay (IGRA) was also negative. Serology for* Brucella abortus* and* melitensis* also came negative. Tests for chronic viral hepatitis B and C were also normal. Serological tests for connective tissue disease were negative as was the serum angiotensin converting enzyme level. Other relevant investigations are outlined in Table 1. An upper abdominal ultrasound showed hypoechoic, cystic lesions scattered across both lobes of the liver (Figure 2). A subsequent CT scan of the abdomen with contrast showed multiple oval hypoattenuating lesions in both lobes of the liver (Figure 3).

**Figure 4:**
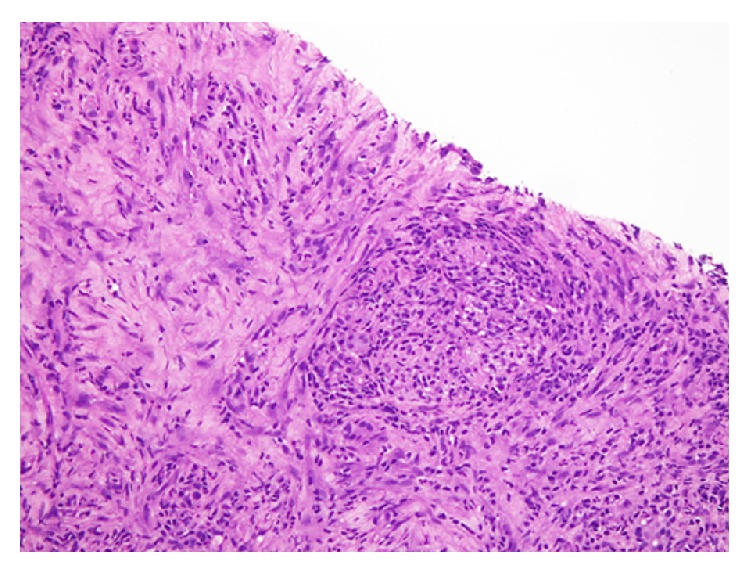
Liver biopsy. Cholestasis with multiple large areas of necrosis palisaded by epitheloid cells with associated granulomatous inflammation.

On liver biopsy there was evidence of cholestasis and multiple large irregular areas of necrosis, palisaded by epitheloid cells with associated granulomatous inflammation with a number of plasma cells (Figure 4).

**Figure 5:**
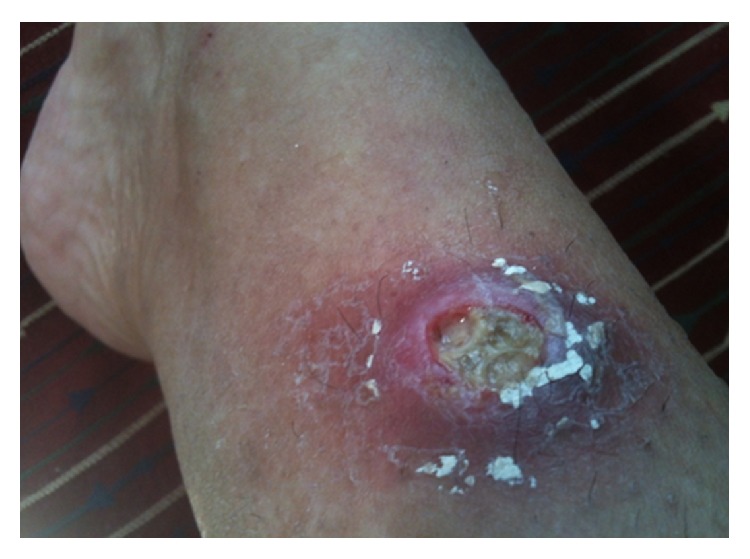
Ulceration in the nodule with erythema and discharge.

**Figure 6:**
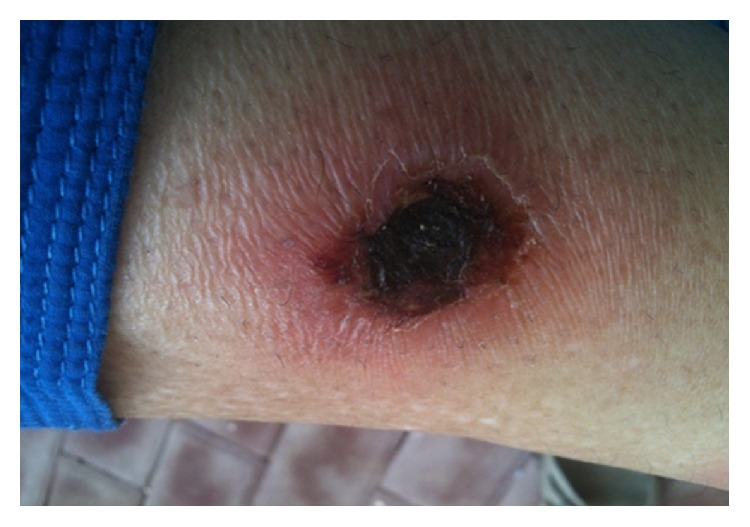
Ulceration progressing to central necrosis.

Acid-fast bacilli culture of liver biopsy specimen after six weeks came out negative. Meanwhile, the patient continued to spike fever with maximum temperature of 101°F. Leg lesions were becoming worse with central necrosis and purulent discharge (Figures 5 and 6).

**Figure 7:**
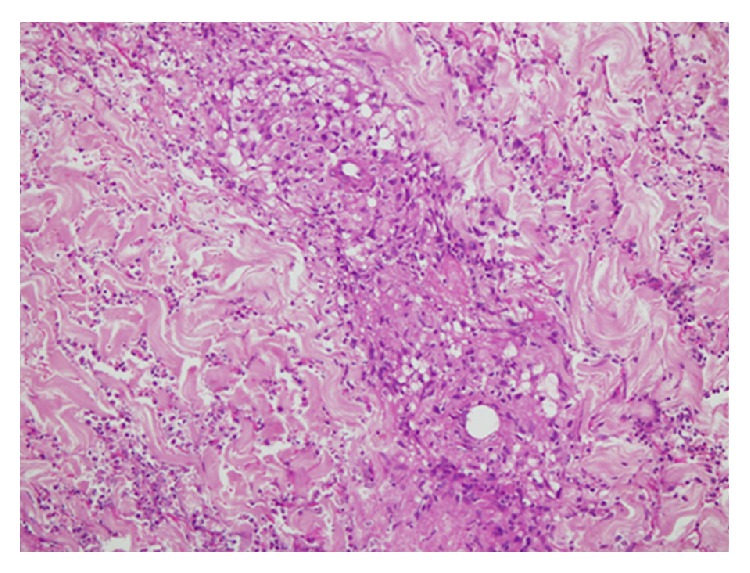
Skin biopsy. Vasculitis involving the superficial and deep dermis showing fibrinoid necrosis of vessel wall with granuloma formation, neutrophilic infiltrate, and nuclear debris.

Pus cultures were negative for bacterial, fungal, and mycobacterial growth. A biopsy of the skin was performed. Pathological examination of frozen sections revealed surface ulceration with fibrinoid necrosis and vasculitis involving superficial and deep dermal vessels along with granuloma formation, neutrophilic infiltration, and debris (Figure 7).

**Figure 8:**
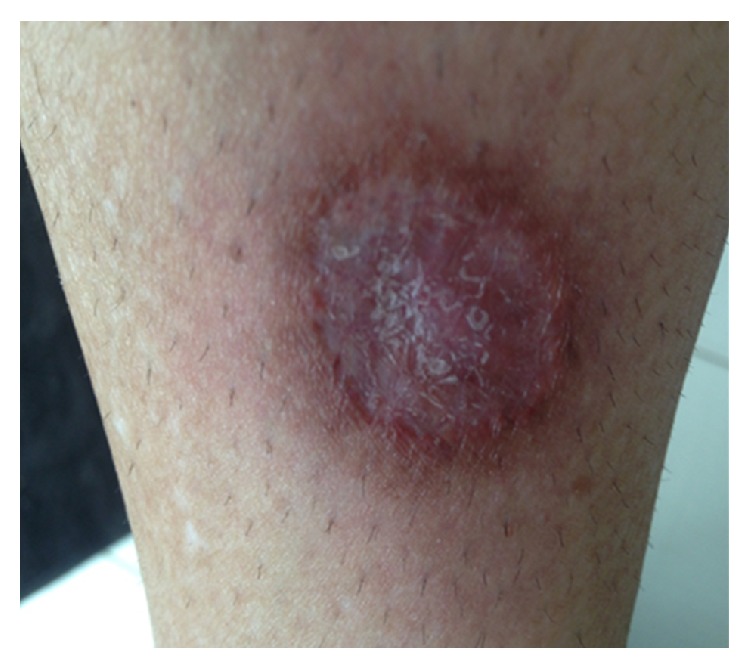
After treatment with 7 weeks of prednisone and azathioprine.

Immunohistochemical testing was performed on biopsy specimen using immune-alkaline phosphatase technique which came negative for mycobacterial species. Real-time PCR for mycobacterial species also turned out to be negative. It was concluded from the skin biopsy that the patient had cutaneous granulomatous vasculitis. Treatment was commenced with prednisone 1 mg/kg/day and the patient was offered induction with cyclophosphamide which she refused due to associated toxicities. Meanwhile, azathioprine 2 mg/kg/day was added to prednisone. After seven weeks on prednisone and six weeks of azathioprine her lesions had healed almost completely (Figure 8). She was maintained on prednisone 5 mg bid and azathioprine 50 mg bid with healing lesions. A repeat CT scan of the abdomen revealed normal architecture of hepatic parenchyma.

Therefore, considering a chronic inflammatory process characterized by hearing loss, gingival hypertrophy, granulomatous hepatic lesions, and cutaneous ulcers with necrotizing granulomatous inflammation on skin biopsy, a unifying diagnosis of granulomatosis with polyangiitis (GPA) was made.

## 3. Discussion

Granulomatosis with polyangiitis (GPA) is complex multisystemic disease with varying manifestations. The 2012 Chapel Hill Consensus conference defines GPA as necrotizing granulomatous inflammation usually involving the upper and lower respiratory tract and necrotizing vasculitis affecting predominantly small to medium vessels [8]. However, it is known to involve other organs with variable frequency either as part of the “generalized” disease or as “limited” or “localized” forms [9]. In this form, the disease does not fulfill the diagnostic criteria early in the course but years later can herald in a severe life threatening multiorgan form [10].

The initial symptoms in our patient were vertigo and decreased hearing in her left ear. Thereafter, she developed fever and respiratory symptoms followed by gingival hypertrophy. Later on she developed cutaneous lesions and persistent fever. Cutaneous involvement in GPA can occur in isolation [11] or as part of a generalized disease. Longitudinal studies of organ involvement in more than 100 patients with GPA have reported frequent skin involvement [4, 5, 12]. The localized or limited presentation of this disease can also appear as cutaneous lesions years before the involvement of other systems (Table 2). Kihiczak et al. [13] described a 36-year-old woman who was initially diagnosed as case of sarcoidosis based on conjunctival and nasal mucosal biopsy. Later on, atypical GPA with granulomatous ulcers was diagnosed, after a long protracted course [13]. In another patient, the disease appeared as recurrent nodular lesions on legs with biopsy suggesting nonspecific inflammation. However ten years later, when pulmonary symptoms developed, a repeat skin biopsy showed granulomatous vasculitis as an underlying cause for both cutaneous and pulmonary disease [14]. In yet another report [15], cutaneous finding included papulonodular acneiform eruption followed by development of necrotic ulcers on forehead, arms, and buttocks. Similar evolution of lesion was also noted in our patient who initially had nodular lesions which later evolved into deep necrotizing ulcers.

With the established role of antineutrophil cytoplasmic autoantibodies or ANCAs in the pathogenesis of disease and the availability of commercially available assays, clinicians have better tools to diagnose this complex disease [16]. ANCA is highly positive in patients with severe generalized disease compared to patients with limited disease [17]. However, commercial ANCA ELISAs have not been standardized and vary in their sensitivity and specificity [18]. Sensitivity of ANCA varies according to the assay used and study design, including differences in disease severity and treatment, with figures varying from 34% to 92% [18]. The ANCA can be negative in limited disease as was the case in our patient. In one study, ANCAs were detectable in only 46% of the patients who present with limited/localized disease [19]. In these patients the disease was confined to upper and/or lower respiratory tract with no clinical signs of vasculitis or as early systemic disease and no other feature of organ threatening vasculitis or as limited disease with localized or early systemic manifestation [19]. Patients with localized disease were also younger with a female predominance [19]. In some patients ANCA can never be detected despite persistent disease. Ettl et al. [20] reported a life threatening form of GPA, in which ANCA remained negative despite a fulminant disease course. Then certainly there are instances where cutaneous GPA was not associated with a positive ANCA [21, 22].

Characteristic findings that aid in establishing the diagnosis include histological evidence of focal necrosis, fibrinoid degeneration, palisading granuloma surrounded by neutrophils and histiocytes, and granulomatous vasculitis with lymphohistiocytic infiltrates involving muscular walls [23]. Nonpalisading foci of granular necrosis or fibrinoid degeneration precede the development of the typical palisading granuloma [24]. Interestingly, our patient presented with both cutaneous and hepatic lesions so she underwent both skin and liver biopsies. With a negative ANCA, the diagnosis in our patient was established on histopathology. Her skin biopsy showed superficial ulceration, vasculitis of superficial and deep dermis comprising of fibrinoid necrosis of vessel wall with granuloma formation and neutrophilic infiltrate and nuclear debris consistent with cutaneous granulomatosis with polyangiitis.

Sections examined from the liver biopsy revealed cholestasis and multiple areas of necrosis palisaded by epitheloid cells and chronic inflammation consistent with granulomatous hepatitis. Liver can rarely become involved in granulomatosis with polyangiitis where it can cause chronic granulomatous inflammation [25–27]. It has been described in autopsy reports of patients with GPA. In the literature search of PubMed database, there is a report describing granulomatous and vasculitic lesions in the liver and spleen [28] in the autopsy whereas the nasal biopsy was negative. The authors emphasized that the biopsy may be negative from typical sites in atypical disease forms. In yet another report [29], vasculitic hepatic lesion caused aneurysmal rupture of hepatic artery. I believe that our patient presented with an atypical, extremely rare presentation with cutaneous and hepatic involvement which remains unexplained with other disease processes. Although cutaneous polyarteritis nodosa can present with nodules and leg ulcers, there is absence of visceral involvement, integrity of muscular arteries in the deep dermal tissue is compromised, and granulomatous inflammation is not a feature [30].

The aim of treatment is to prevent the morbidity and mortality associated with the disease and is usually done by remission induction and remission maintenance [4]. Oral cyclophosphamide (CYC) in combination with high-dose glucocorticoids has long been the criterion standard. Due to the toxicities associated with cyclophosphamide (CYC), notably sterility and bladder cancer, Rituximab combined with high-dose glucocorticoids represents an alternative for induction of remission. It is a chimeric monoclonal anti-CD20 IgG1 antibody that induces apoptosis of B cells. For localized disease, methotrexate can be substituted for CYC and added to high-dose steroids, for example, prednisone 1 mg/kg/day for induction of remission. Azathioprine, leflunomide, and methotrexate are all agents for maintenance of remission which should be continued for at least 18 months. We recommended Rituximab to our patient for remission induction as she had refused CYC. While she awaited medical insurance cover for the former, treatment was commenced with prednisone 1 mg/kg/day and azathioprine at a dose of 2 mg/kg/day with remarkable resolution of the lesions after 7 weeks of therapy (Figure 8).

Untreated granulomatosis with polyangiitis is uniformly fatal with mortality of up to 90% at 2 years [4]. Although remission is generally maintained, relapses are common and mortality remains high. A study on long term outcome of GPA patients reported a survival time of 8.5 years [31]. It is therefore important to identify this disease even with limited presentation so that appropriate treatment can be initiated and tailored for an individual patient. Mimics of cutaneous granulomatous vasculitis should be thoroughly investigated and excluded. Where ANCA is negative, tissue biopsy should be obtained to arrive at the diagnosis.

Although numerous classification criteria have been developed to aid in prompt diagnosis and management, there are always instances whereby these diseases can present in an atypical way. The case report that we have highlighted will prompt clinicians to consider the limited and localized forms of granulomatosis with polyangiitis (GPA) so that the disease is recognized early and treated effectively.

## Figures and Tables

**Table 1:** Laboratory data.

Investigations	Results
Hb/Hct	10.5/35
WBC	8.5 × 10^9^/L
Platelet count	226 × 10^9^/L
Blood urea nitrogen	12 mg/dL
Serum creatinine	0.8 mg/dL
Total bilirubin (direct/indirect)	0.7 mg/dL (0.5/0.2)
Alanine aminotransferase	13 U/L
Alkaline phosphatase	107 U/L
Urinalysis	pH: 5.5, no glucose/ketones/casts/protein, WBCs 3-4/high power field
Blood cultures (3 sets)	No growth
Urine culture	No growth
Interferon gamma release assay (IGRA)	Negative
Brucella abortus and melitensis titres	<1 : 80 ×2
Hepatitis B surface antigen (HBsAg)	Negative
Anti-HCV antibody	Negative
Antinuclear antibody	Negative
Anti-dsDNA	Negative
ACCP antibody	Negative
QRA	27.7 (elevated)
c-ANCA	Negative (0.8 U/mL)
p-ANCA	Negative (1.86 U/mL)
Angiotensin converting enzyme	40 U/L (normal <52 U/L)

**Table 2:** Case reports of localized GPA with cutaneous manifestations.

Author and year of publication	Diagnosis	Organ involvement	ANCA	Clinical course
Kihiczak et al. [13] 1994	Protracted superficial GPA	Skin, nasal mucosa	N/A	Protracted course characterized by granulomatous skin and nasal ulcers

Figarella et al. [14] 2000	Protracted superficial GPA	Upper respiratory tract, skin, and lung	N/A	Skin nodules on leg present for 10 years, before the diagnosis was established based on cutaneous histopathology

Brazzelli et al. [32] 1999	Cutaneous GPA	Acneiform nodular and popular eruption on forehead, dense pulmonary infiltrates	Positive	Necrotic ulcers on forearms, arms, and buttocks, small vessel vasculitis of deep dermis

Kuchel and Lee [21] 2003	Cutaneous GPA	Skin involving ear lobe, ankles, and feet	Negative	Responded to prednisone and CYC, granulomatous inflammation on biopsy

Sinovich and Snow [33] 2003	Protracted superficial GPA	Infra-auricular skin and nasal mucosa	Positive	Pyoderma gangrenosum like lesions with suppurative granulomatous inflammation. Responded to prednisone and azathioprine

Kawakami et al. [34] 2005	GPA with cutaneous leukocytoclastic vasculitis	Skin, lung, and kidney	Positive	Various cutaneous manifestations. Palisading neutrophilic granulomatous dermatitis on histopathology

Ben Ghorbel et al. [35] 2007	Cutaneous and mucosal GPA as initial presentation in an adult	Tongue and labial ulcers, digital necrosis, splinter hemorrhages of fingernails, and purpura	Positive	Diagnosis confirmed on cutaneous and renal biopsies. Resolution of cutaneous disease after 6 weeks of treatment with prednisone and CYC

Ben Ghorbel et al. [15] 2007	Recurrent digital necrotic ulcers on fingers, toes, and scapula as cutaneous GPA in a child	Nasal mucosa, eyes (keratitis), and skin	Positive	Superficial ulcerations with granulomatous vasculitis

Thaiwat and Aunhachoke [22] 2010	Superficial GPA with chronic scalp ulcer	Skin	Negative	Solitary nodule on scalp with alopecia; nodules on left arm and shin; purpura
